# Production of high loading insulin nanoparticles suitable for oral delivery by spray drying and freeze drying techniques

**DOI:** 10.1038/s41598-022-13092-6

**Published:** 2022-06-15

**Authors:** Yigong Guo, Alberto Baldelli, Anika Singh, Farahnaz Fathordoobady, David Kitts, Anubhav Pratap-Singh

**Affiliations:** 1 Faculty of Land and Food Systems (LFS), The University of British Columbia, Vancouver Campus 213-2205 East Mall, Vancouver, BC V6T 1Z4 Canada; 2 Natural Health and Food Products Research Group, Centre for Applied Research and Innovation (CARI), British Columbia Institute of Technology, 4355 Mathissi Pl, Burnaby, BC V5G 4S8 Canada

**Keywords:** Biological techniques, Biophysics, Biotechnology, Drug discovery, Environmental sciences, Diseases, Medical research, Energy science and technology, Engineering, Materials science, Nanoscience and technology, Physics

## Abstract

Insulin nanoparticles (NPs) with high loading content have found diverse applications in different dosage forms. This work aimed to evaluate the impact of freeze-drying and spray drying process on the structures of insulin-loaded chitosan nanoparticles, with or without mannitol as cryoprotectants. We also assessed the quality of these nanoparticles by redissolving them. Before dehydration, the chitosan/sodium tripolyphosphate/insulin crosslinked nanoparticles were optimized to 318 nm of particle size, 0.18 of PDI, 99.4% of entrapment efficiency, and 25.01% of loading content. After reconstitution, all nanoparticles, except the one produced by the freeze-drying method without using mannitol, maintained their spherical particle structure. The nanoparticles dehydrated by spray drying without mannitol also showed the smallest mean particle size (376 nm) and highest loading content (25.02%) with similar entrapment efficiency (98.7%) and PDI (0.20) compared to mannitol-containing nanoparticles dehydrated by either spray drying or freeze-drying techniques. The nanoparticles dried by spray drying without mannitol also resulted in the fastest release and highest cellular uptake efficacy of insulin. This work shows that spray drying can dehydrate insulin nanoparticles without the need for cryoprotectants, creating a significant advantage in terms of greater loading capacity with lower additive requirements and operating costs as compared to conventional freeze drying approaches.

## Introduction

Insulin and its pharmaceutical formulations have saved the lives of type I diabetic Mellitus (T1DM) and type II diabetic Mellitus (T1DM) patients since its discovery in 1922^1–3^. However, due to its property as a high-molecular-weight protein, insulin is readily aggregated, decomposed by the proteolytic enzymes, and eliminated by the first-pass effect^4^. Patients diagnosed with type I diabetes are injected with insulin for the rest of their lives. Many patients diagnosed initially with type 2 diabetes also require insulin injections in the long term. Injecting insulin daily is a grave source of daily pain and discomfort for these individuals, negatively affecting mental health. As a result, other modes of insulin delivery that cause less discomfort, like oral insulin administration, are being extensively researched^5^, as they have the potential to bring the quality of life back to about half a billion diabetic patients around the world.

Nanoparticle technology offers significant advances while trying to deliver insulin orally^4,6,7^. One can efficiently encapsulate and protect insulin from degradation for targeted deliveries to specific human body parts. However, the usage of nanoparticle formulations has several limits, mainly due to issues with the stability of the particle suspension. Some aggregation may occur during storage, which can decrease the bioavailability of the insulin-loaded nanoparticles^8^. In addition, the chemical stability of the polymeric matrix of the nanoparticles and insulin must also be considered to ensure the stability of the insulin nanoparticles (NPs). Currently, freeze-drying technology is the gold standard for creating stable NPs, while preventing unwanted changes during storage^9^.

Nevertheless, freeze-drying requires the addition of cryoprotectants to prevent the spherical structures of the NPs from the mechanical stress of ice crystals^9^. This significantly decreases the loading content of the insulin NPs after freeze-drying since the cryoprotectants occupy most of the weight ratio. Thereby, produced insulin NPs are often found unsuitable for creating dry powdered formulations such as oral tablets and oral films since vast amounts of dry nanoparticles are required to achieve the therapeutic window of the insulin.

Spray drying is a well-known and inexpensive industrial-scale process for generating a dry powder from a liquid phase in the pharmaceutical industry^10,11^. The control over the particle formation process allows a proper encapsulation of several bioactive compounds^12,13^. In addition, it has been an effective technique for preparing encapsulated proteins for oral administration^11^. During spray drying, the evaporation rate of water is very fast, which helps keep the temperature of the particle core low^11,14^, thus allowing for its’ application in encapsulating heat-sensitive ingredients. Before spray drying, the coating material should be thoroughly homogenized with the solution containing encapsulated components^11,14^. Unlike freeze-drying, homogenization before spray drying encapsulation enhances the encapsulation efficiency during the dehydration process. As cryoprotectants are not necessary for the spray drying encapsulation process, and thus, spray drying can be used to produce dry NPs with high loading content.

This study reports the process for producing insulin-loaded NPs by creating a crosslink of chitosan and sodium tripolyphosphate using the ionotropic gelation method. Ionotropic gelation is a preparation method that allows the production of nanoparticles via electrostatic interactions between two or more ionic species under certain conditions. Both freeze-drying and spray drying techniques were utilized to dehydrate optimized chitosan/sodium tripolyphosphate/insulin crosslinked NPs. After dehydration, their morphologies were analyzed by SEM. Their reconstitution capacity was evaluated by measuring their size distribution, surface charge, PDI, encapsulation efficiency, and loading content. The quality of the redissolved nanoparticles produced by different dehydration methods was also evaluated by comparing their insulin protection effects, release behaviors and cellular uptake efficacy.

## Results and discussion

### Optimization of different parameters to generate homogenized insulin NPs

**Figure 1:**
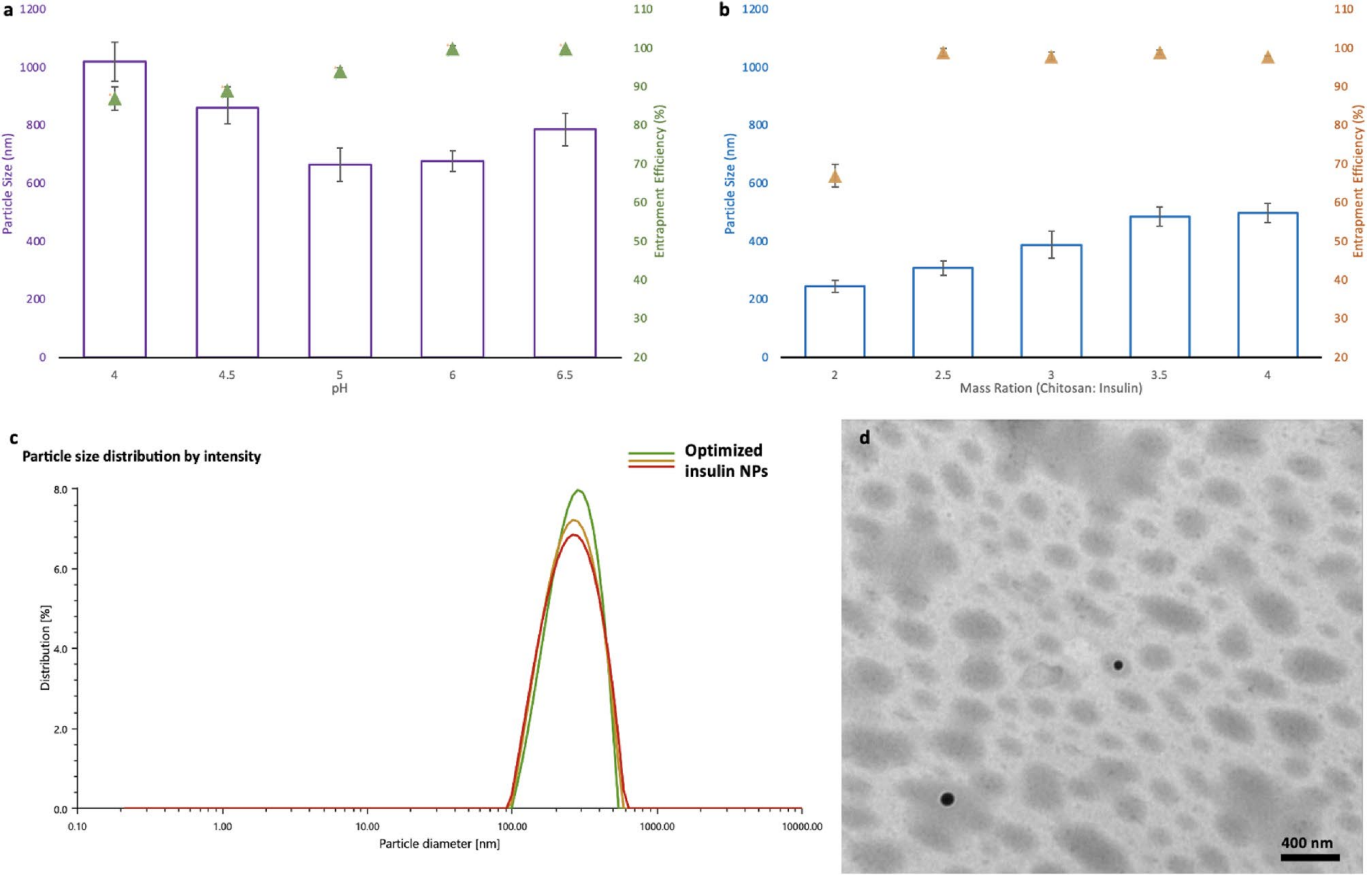
Optimization of parameters for insulin NPs: (**a**) Effect of the pH on the average diameter and the encapsulation efficiency (EE) of the insulin NPs (prepared under the mass ratio of chitosan and insulin at 5:1); (**b**) Effect of the mass ratio between chitosan and insulin the average diameter and the encapsulation efficiency (EE) of the insulin NPs (prepared at pH 6); (**c**) Particle size distribution of the optimized insulin NPs; (**d**) TEM micrographs of the optimized insulin NPs.

The pH of the mixing solution and the ratio of the chitosan and insulin were two critical factors affecting the particle size and encapsulation efficiency (EE) of the final NPs, as they directly influenced the ionotropic gelation process^15^. The pH of the mixing solution was shown to have high associations with both particle size and encapsulation efficiency (Fig. 1a). As shown in Fig. 1a, the mean particle size (nm) dropped, and EE increased remarkably with pH increased from 4.0 to 6.0, while the mean particle size started to increase and the EE remained the same once the pH increased to 6.5. With increasing the ratio of chitosan to insulin, the mean particle size kept increasing as well. Besides, no change in the EE was observed when the nanoparticles were prepared under a chitosan/insulin mass ratio higher than 2.5:1 (w/w) (Fig. 1b). Accordingly, the optimal preparing condition in this research (pH 6.0 and chitosan/insulin mass ratio of 2.5:1) was used to prepare insulin-loaded nanoparticles for further studies. With this preparation condition, the insulin NPs were optimized to 318 nm of mean particle size (Fig. 1c), 0.18 of PDI, 99.4% of entrapment efficiency, 9.8 mv of zeta potential 25.01% (m/m) of insulin loading content. Based on the transmission electron microscope (TEM) results, the optimized nanoparticles were roughly spherical and discrete with relatively uniform size (Fig. 1d).

It is well known that chitosan is a weak polyelectrolyte with a pKa of 6.5. It is positively charged in acidic media because its main amino groups are protonated by hydrogen ions^15^. It is, therefore, frequently utilized as a carrier for encapsulation of negatively charged macromolecules. In this study, chitosan was used to encapsulate insulin with an isoelectric point of 5.3. Since the chitosan served as a coating material, with the increase of its ratio, the thickness of the outer layer of the NPs increased accordingly, resulting in a larger mean particle size. Additionally, higher content of chitosan can encapsulate more insulin. In our case, the highest EE was achieved once the ratio of chitosan and insulin reached 2.5:1, and no significant change in the EE was observed when the ratio kept increasing.

Apart from the chitosan and insulin ratio, pH also played an essential role in NPs preparation. Gan et al.^17^ investigated the influence of pH on chitosan NPs particle size. They found that the particle size showed a continuous decrease before the pH reached the value of 6.0, and a significant increase in size was observed at pH > 6.0, which agrees with our observations. This phenomenon is because as pH rises, insulin molecules gain a negative surface charge, thus, favoring electrostatic interactions with the chitosan/ Sodium tripolyphosphate (TPP) complex and resulting in small particle sizes and high EE. However, when pH was tuned to 6.5, deprotonation of amino groups on chitosan occurred, leading to the folding of chitosan^18^. Thus, high pH caused fewer amino ions to be exposed to TPP and insulin, resulting in low crosslinking and eventually larger mean particle size and low EE.

### Characterization of the dehydration NPs

#### Morphology analysis

**Figure 2:**
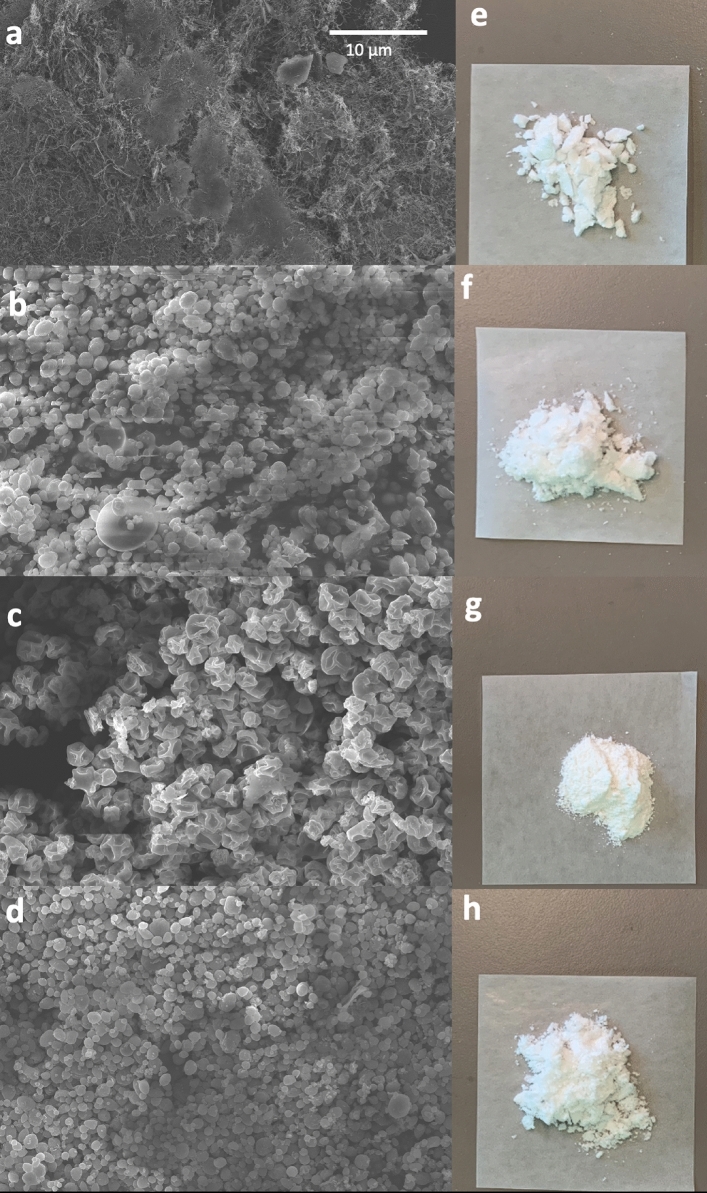
Morphology of different dehydrated insulin NPs: (**a**) SEM image of freeze-dried insulin NPs without mannitol; (**b**) SEM image of freeze-dried insulin NPs with mannitol; (**c**) SEM image of spray-dried insulin NPs without mannitol; (**d**) SEM image of spray-dried insulin NPs with mannitol; (**e**) Image of freeze-dried insulin NPs powder without mannitol; (**f**) image of freeze-dried insulin NPs with mannitol; (**g**) Image of spray-dried insulin NPs powder without mannitol; (**h**) Image of spray-dried insulin NPs powder with mannitol.

Analyzing the morphological properties of freeze-dried and spray-dried NPs can guide selecting better dehydration and powder formation techniques. The preferred approach should provide drug stability, uniform particles shape, high drug loading, and good solubility in the original solution. In this study, to better compare the two techniques, insulin NPs with or without 1% of mannitol were used for the dehydration process. Mannitol was used as a bulking agent or cryoprotectants in various dry powder formulations for freeze-drying and spray drying. For the freeze-dried insulin NPs with no mannitol, as shown in Fig. 2a, a highly porous powder structure with large, irregular, and rough surfaces was observed under the scanning electron microscope (SEM). Almost no discrete particles could be detected in the powder after dehydration (Fig. 2e). These results indicated that most NPs were broken down during freeze-drying without any cryoprotectants. For both freeze-dried and spray-dried insulin NPs with 1% of mannitol, a spherical shape with a smooth surface of NPs was observed (Fig. 2b, d, f, h). The insulin NPs spray-dried with no mannitol kept their spherical shape but had a wrinkled surface (Fig. 2c). The spherical and wrinkled surfaces were further discussed in the following release behavior and cell uptake tests. Based on the visible appearance of the dry NPs, the NPs spray-dried with no mannitol and NPs freeze-dried and spray-dried with mannitol were all resulted in fine NPs powders (Fig. 2f, g, h). Higher surface area between the surface of microparticles induces a higher solubility and, thus, a higher release rate^19^.

During freeze-drying, mannitol acted as a cryoprotection agent, remaining in amorphous forms and preventing the NPs from damage by the ice crystal^19^. In contrast, there is no freezing step during the spray drying process. The mannitol was therefore not necessary in this method. In fact, as discussed earlier, spray-dried NPs without mannitol produced finer NPs. However, the mannitol could still serve as a bulking agent in the spray drying process to impart NPs a more spherical structure^20^ (Fig. 2d), which could help obtain a uniform release behavior of such encapsulated NPs. Moreover, it was evident that some large particles can be detected in both freeze-dried and spray-dried insulin NPs with mannitol (Fig. 2b, d), which probably resulted from the accumulation of mannitol in the particle's core alongside insulin inside the encapsulating layer of chitosan. Noticeably, in this study, to make sure that the spherical structure can remain intact after the dehydration, the ratio of mannitol and chitosan was kept at 5:1 so that the high amount of bulking agent can also enlarge the particle size of the dry NPs.

#### FTIR analysis

**Figure 3:**
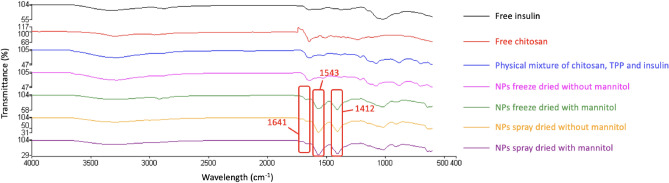
FTIR-ATR spectra of free insulin, chitosan, physical mixture of chitosan/TPP/insulin and NPs dehydrated by different methods.

Fourier Transform Infrared-attenuated total reflectance (FTIR-ATR) spectroscopy characterized free insulin, chitosan, a physical mixture of chitosan, TPP, and insulin. All dehydrated NPs were characterized by using FTIR-ATR spectroscopy. Noticeably, band intensities at 1641, 1543, and 1412 cm^−1^ were observed in encapsulated NPs freeze-dried with mannitol and NPs spray-dried both with and without mannitol (Fig. 3). These increases in intensities are associated with the cross-link among chitosan, TPP, and insulin, as previously reported^21^. Studying the interaction between chitosan and insulin indicated that, in the FTIR spectra of insulin-loaded chitosan NPs, the chitosan bands overlapped with those of insulin, increasing the carbonyl intensity (1641 cm^−1^) and amine (1543 cm^−1^) bands. The tripolyphosphate groups of TPP linked with ammonium groups in chitosan caused the band at 1412 cm^−1^.

Furthermore, these results were consistent with the results shown in SEM, which indicated the encapsulated NPs remained intact for both spray and freeze-drying with mannitol, but without mannitol, only spray drying could yield encapsulated particles. In contrast, the FTIR-ATR spectra result of the NPs freeze-dried without mannitol was very similar to the physical mixture of chitosan, TPP and insulin. This result indicated that cross-link among chitosan, TPP, and insulin no longer existed in NPs freeze-dried without mannitol. The NPs structure was broken during freeze-drying without cryoprotectant, which could be seen in the SEM result (Fig. 2a). Based on the morphological and FTIR results of the dehydrated insulin NPs, as the NPs freeze-dried with no mannitol broke down during the dehydration process, only NPs freeze-dried with mannitol, spray-dried with and without mannitol were used for reconstitution tests and discussion.

#### Reconstitution and stability analysis

Dehydration is being used for long-term storage and reprocessing to other formulations. The reconstitution ability of the dry NPs after the storage is critical for their use in different formulations such as tablets and films. We noticed that the mean particle size of the insulin NPs spray-dried with no mannitol was only slightly increased after reconstitution. On the other hand, the size of particles of insulin NPs spray-dried and freeze-dried with mannitol increased significantly (Table 1). The PDI and EE did not change substantially (*p* > 0.05) after the reconstitution for all NPs in this study (Table 1). This result indicated that most of the particles remained intact after redissolved. However, adding mannitol caused the insulin loading content of freeze-dried and spray-dried NPs with mannitol to decrease tremendously (Table 1). In contrast, the insulin loading content of NPs spray-dried without mannitol remained the same as before (Table 1).

**Table 1:** Physicochemical properties of freshly prepared and reconstituted NPs (one way ANOVA comparisons, *p* < 0.05).

	Freshly prepared NPs	NPs freeze-dried with mannitol	NPs spray dried without mannitol	NPs spray-dried with mannitol
Z-average diameter (nm)	318 ± 18^a^	530 ± 67^b^	376 ± 47^c^	578 ± 37^b^
Polydispersity index	0.18 ± 0.01^a^	0.23 ± 0.05^a^	0.20 ± 0.03^a^	0.22 ± 0.05^a^
Encapsulation efficiency (%)	99.40 ± 0.43^a^	98.74 ± 0.54^a^	98.67 ± 0.39^a^	99.13 ± 0.31^a^
Loading content (%)	25.22 ± 0.17^a^	5.67 ± 0.13^b^	25.02 ± 0.34^a^	5.38 ± 0.26^b^

It is well known that nanoparticles’ loading content is crucial when applied for drug delivery purposes. For NPs with low loading content, a very high quantity of material is required to achieve the therapeutic threshold. However, the high viscosity of such high NP concentrations leads to inconvenience and difficulties for oral delivery and injection formulations, respectively^22^. In addition, insulin NPs can also be used to make tablets and adhesive biofilms^23,24^, necessitating using a huge amount of low loading content NPs, resulting in large tablets and thick biofilms inappropriate for oral application. Accordingly, dehydrated NPs with high insulin loading are highly demanded. Our results suggested that the high insulin loading of the spray-dried NPs without mannitol could offer many attractive advantages to these alternative administration methods.

**Figure 4:**
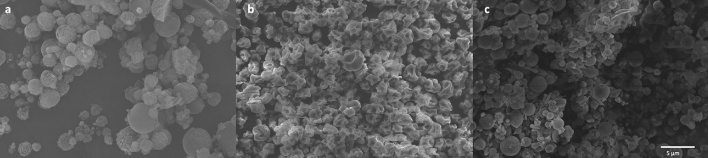
Morphology of different dehydrated insulin NPs stored for three months: (**a**) SEM image of freeze-dried insulin NPs with mannitol; (**b**) SEM image of spray-dried insulin NPs without mannitol; (**c**) SEM image of spray-dried insulin NPs without mannitol.

All dehydrated NPs were stored in the fridge for three months. The SEM result indicated that the morphology of all the dehydrated NPs had no visible changes during the three-month storage (Fig. 4). After reconstitution in water, The EE of all the NPs slightly decreased, and around a small amount (~5%) of insulin was released during the three-month storage (Table 2). However, the mean particle size of all the NPs increased. The particle size of the NPs spray dried without mannitol was increased to 525 nm while the particles size of the spray-dried and freeze-dried NPs with mannitol increased to 872 and 921 nm, respectively (Table 2).

**Table 2:** Physicochemical properties of NPs after three-month storage (one way ANOVA comparisons, *p* < 0.05).

	NPs freeze dried with mannitol	NPs spray dried without mannitol	NPs spray dried with mannitol
Z-average diameter (nm)	921 ± 102^a^	525 ± 77^b^	872 ± 117^c^
Polydispersity index (PDI)	0.25 ± 0.07^a^	0.23 ± 0.02^a^	0.26 ± 0.07^a^
Encapsulation efficiency (%)	91.36 ± 0.73^a^	92.00 ± 0.88^a^	92.29 ± 0.67^a^
Loading content (%)	5.06 ± 0.32^a^	23.02 ± 0.41^b^	5.08 ± 0.22^a^

Moreover, precipitates could be seen in the redissolved insulin NPs spray dried and freeze-dried with mannitol (Fig. S2). This was probably caused by the big particles, which could not suspend in water properly. All the results above showed that the spray drying technique can protect the insulin NPs from dehydration, and the high loading content of insulin NPs without any bulking agent or cryoprotectants could be obtained.

### Insulin protection effects

**Figure 5:**
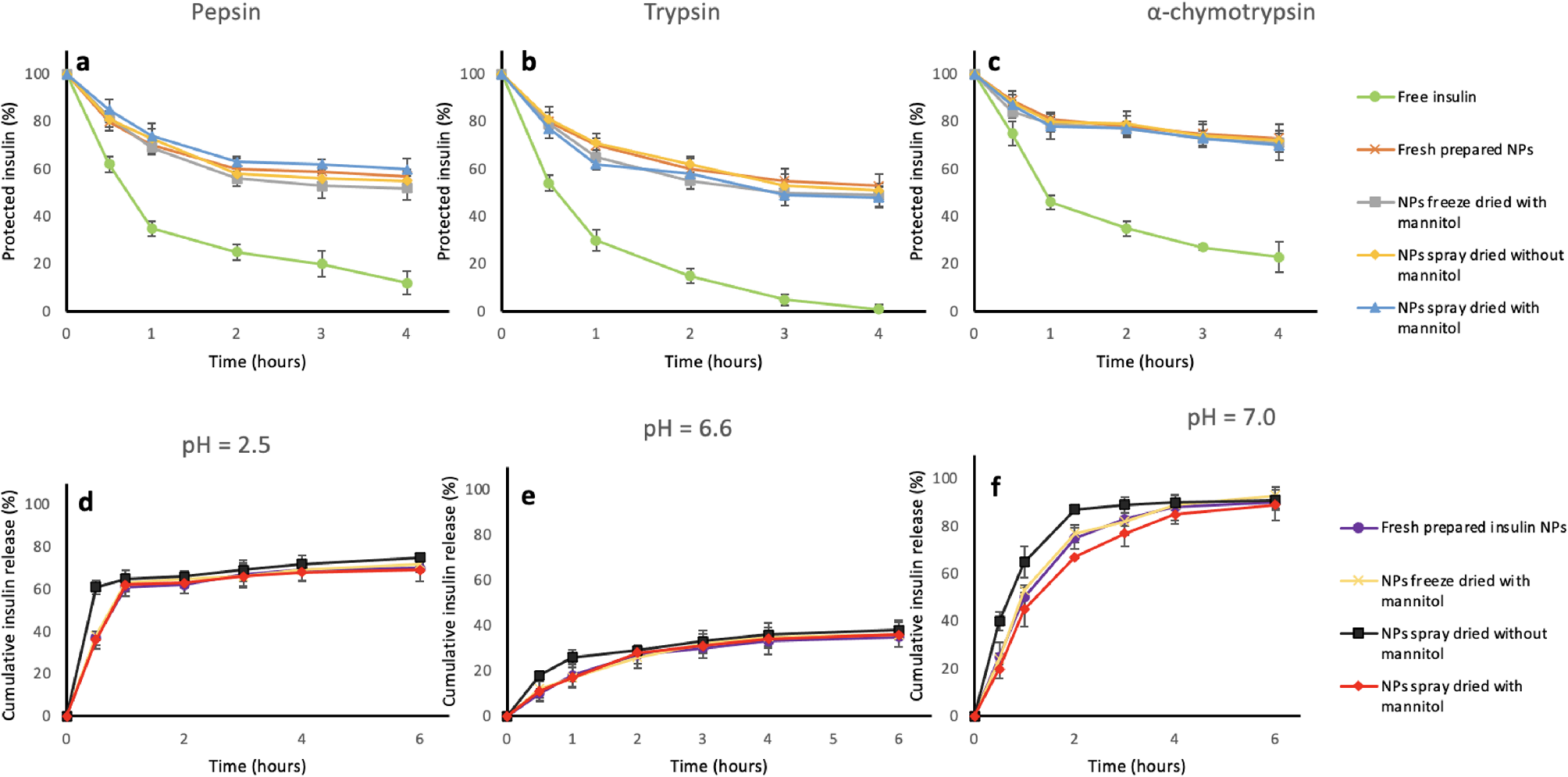
Protection effects and release behaviors of dehydrated insulin NPs: (**a**) Protection of insulin at pepsin solution; (**b**) Protection of insulin at trypsin solution; (**c**) Protection of insulin at α-chymotrypsin solution; (**d**) Release behaviors of dehydrated NPs in pH = 2.5 solutions; (**e**) Release behaviors of dehydrated NPs in pH = 6.6 solutions; (**f**) Release behaviors of dehydrated NPs in pH = 7.0 solution.

Insulin retention ratio was tested in pH = 2.5 medium with pepsin, trypsin, and α -chymotrypsin to demonstrate the protection capacity of the NPs against enzymatic digestion after dehydration. The insulin retention ratio of the dehydrated NPs was compared to the freshly prepared NPs, and the free insulin was used as the negative control. In this study, free insulin showed fast insulin elimination within 4 h in all three enzyme treatments (Fig. 5a–c). In contrast, the insulin elimination test of the NPs freeze-dried with mannitol and the NPs spray-dried both with or without mannitol exhibited significantly higher protection effects of these NPs against enzyme digestion, which was similar to the freshly prepared insulin NPs (Fig. 5a–c). More than 50%, 60%, and 75% of the insulin can be protected within 4 h with the help of nanoparticles in pepsin, trypsin, and α -chymotrypsin, respectively (Fig. 5a–c). This insulin protection ability can increase the opportunity for higher insulin absorption into the blood circulation^25^. These results suggest that spray drying with or without mannitol and freeze-drying with mannitol can maintain the insulin protection ability of the NPs after dehydration.

### In vitro release of insulin from NPs at different pH conditions

Freshly prepared and redissolved dry insulin NPs were incubated in various buffers (pH = 2.5, 6.6, 7.0) at 37 °C, modeling the pH environment in the stomach, duodenum, and upper small intestine, to examine the release behaviors of insulin in different segments of the GI tract. At pH = 2.5, insulin-loaded NPs and redissolved dry insulin NPs showed an initial burst release in the first one hour, followed by a slow release in the next 5 h (Fig. 5d). This fast release at the beginning was most probably the result of rapid surface desorption of protein molecules that were not fully entrenched in the particle's interior structure. At pH = 6.5, insulin-loaded NPs and redissolved dry insulin NPs showed a smoothly slow release in 6 h because the pH of the testing solution was similar to the NPs preparation solution (Fig. 5e). At pH = 7, NPs were unstable and almost fully disintegrated in the first two hours (Fig. 5f). This resulted from the fact that the deprotonation of chitosan happened at higher pH, which caused a less compact polymer network and the release of the loaded insulin.

Furthermore, the insulin NPs spray-dried with no mannitol showed a faster release profile than other dehydrated NPs (Fig. 5d–f). As previously discussed, the redissolved insulin NPs pray dried with no mannitol showed the smallest particle sizes. The small particles provided a large surface area so that most of the drugs associated would be at or close to the particle surface, resulting in fast drug release^26^.

### In vitro toxicity evaluation

An MTT assay was employed to investigate the cytotoxicity of NPs. As shown in Fig. S4, all dehydrated NPs were found to have no significant impact on the cell viability at the concentration of 50–500 μg/ml, which indicated that all dehydrated NPs can be safely used to reach the therapeutic window.

### In vitro cellular uptake of dehydrated NPs

**Figure 6:**
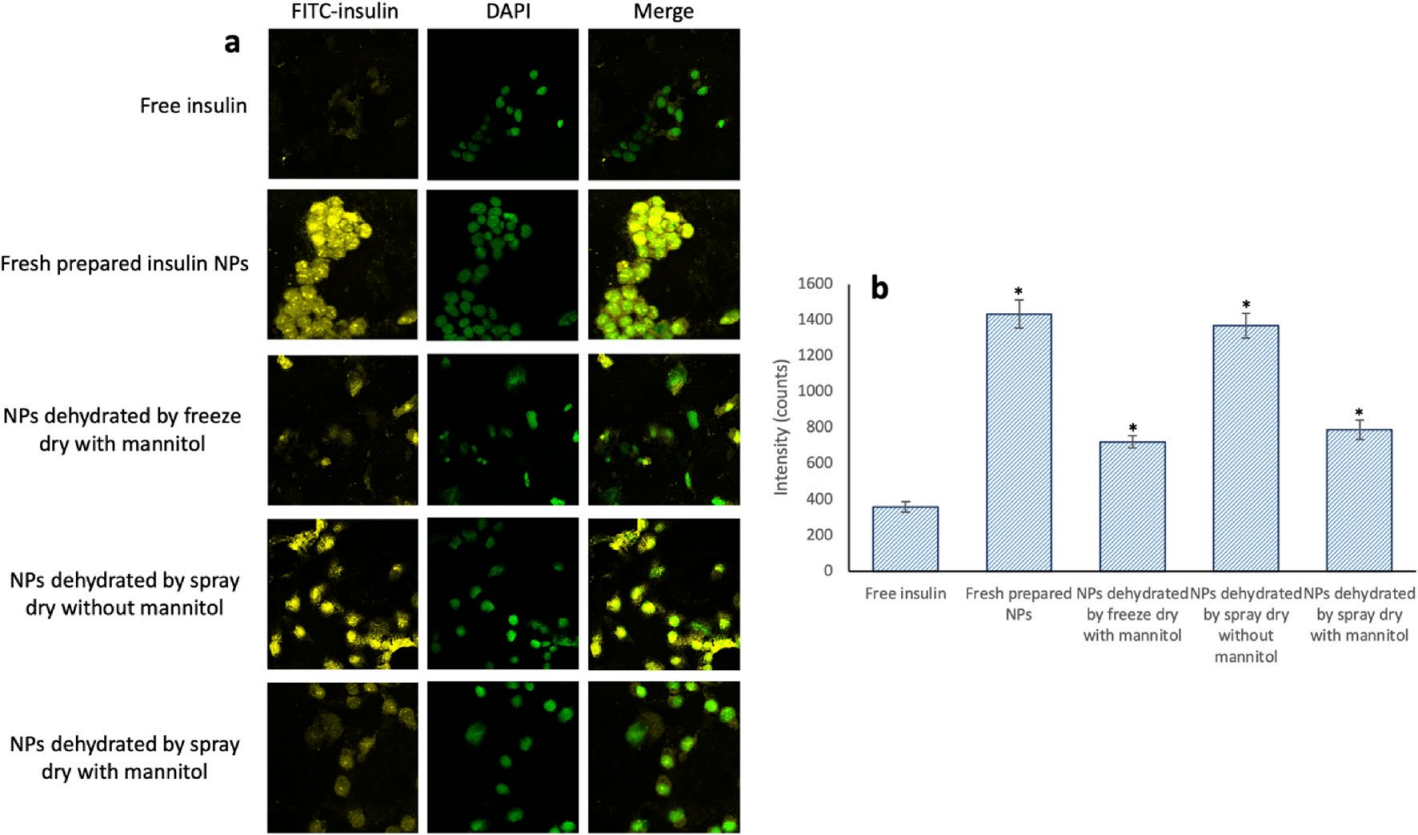
HepG2 cellular uptakes after 4 h incubation with fresh prepared NPs and dehydrated NPs: (**a**) Distribution of FITC-insulin uptaken by HepG2 cells. (**b**) Geometric mean values of the fluorescence intensities of the flow cytometry analysis (*n* = 3), *P < 0.05 compared with free insulin.

The liver is the primary organ where insulin performs its physiological function. HepG2 cell is a human liver cancer cell line commonly utilized as a hepatocyte absorption model in vitro. Herein, HepG2 cell was used to evaluate the cellular uptake of NPs dehydrated using freeze-drying and spray-drying methods. After hours of incubation with free FITC-insulin concentration of 25 μg/mL, equivalent insulin concentrations of freshly prepared FITC-insulin loaded NPs, and dehydrated FITC-insulin loaded NPs, the cellular uptakes were quantified using flow cytometry and visually by confocal laser scanning microscopy (CLSM) observation. The freeze-dried NPs without mannitol were broken during the dehydration process and were not evaluated in this test. The intracellular fluorescence intensities (Fig. 6a) of freshly prepared insulin-loaded NPs, freeze-dried NPs with mannitol, and spray-dried NPs with and without mannitol were 4.3, 2.6, 2.4 and 4.1-fold higher than the intensity of the free FITC-insulin group, respectively (Fig. 6b). These results demonstrate the higher efficacy of encapsulated insulin than free insulin in cellular uptake, which is primarily associated with the smaller size of the insulin-loaded NPs created in the study.

Similarly, the CLSM images showed that the FITC fluorescence intensity of fresh prepared FITC-insulin loaded NPs and FITC-insulin loaded spray-dried NPs without mannitol was much stronger than the intensities of the other samples (Fig. 6a). Besides, with the addition of mannitol, the higher viscosity of the solution increased the resistance of cellular uptake^27^, resulting in lower insulin proliferation. These results indicate that spray-dried NPs without mannitol demonstrated the highest cellular uptake efficiency, as their particle sizes were smaller than freeze-dried NPs once redissolved.

## Materials and methods

### Materials

Chitosan (Average Mw 100 KDa, 75–85% deacetylated) was purchased from Sigma-Aldrich. (Oakville, Ontario, Canada). Sodium tripolyphosphate (TPP) was purchased from VWR (Radnor, Pennsylvania, USA). Recombinant human insulin used in this study was from Fisher Scientific (Waltham, Massachusetts, USA). Fluorescein isothiocyanate (FITC)-labelled human insulin and the 4’, 6-Diamidino-2-phenylindole dihydrochloride (DAPI) were purchased from Sigma-Aldrich. (Oakville, Ontario, Canada). The HepG2 cell line was obtained from ATCC (Manassas, Virginia, USA). All the other reagents were of analytical or chromatography grade.

### Insulin nanoparticles (NPs) preparation

One mg/ml of CS solution was prepared by dissolving it in double-distilled water (DD water) containing 0.1% acetic acid. 1 mg/ml of TPP and insulin solution were prepared by dissolving them in DD water and 0.1% acetic acid, respectively. The pre-emulsions were prepared by a polytron PCU-2-110 high-speed homogenizer (Brinkmann Ind. Westbury, NY, USA). The preparation process is described as follows: 2 ml of TPP solution was first introduced to 4 ml of insulin solution and stirred for 30 min for thoroughly mixing. Then, the mixed solution was added dropwise into CS solution by syringe under high-speed stirring (10,000 rpm). The mixture was maintained under high-speed stirring (15,000 rpm) in the ice bath for 30 min to obtain crosslinked insulin NPs after adjusting them to a certain pH. To further homogenize and decrease the particle sizes of the insulin NPs, they were ultrasonicated for another 30 min in the ice bath using a probe type ultrasonicator (UP 200ST, Hielscher Ultrasonics, Teltow, Germany).

### Characterization of nanoparticles

The Z-average diameter, polydispersity index (PDI) and zeta potential of insulin NPS were tested using dynamic light scattering (DLS) measurements using Litesizer 500 (Anton Paar, Graz, Austria) by diluting them in DD water at 25 °C. Morphology and size distribution was characterized by Hitachi H7600 Transmission electron microscopy (TEM) (Hitachi, Tokyo, Japan), and the images were later analyzed using Hitachi imaging software (Hitachi, Tokyo, Japan). To evaluate the encapsulation efficiency (EE) and loading content (LC) of insulin NPs, the NPs were pipetted into the ultrafiltration tube with a molecular weight cutoff at 100 kDa, followed by centrifugation at 500xg for 30 min. The unencapsulated insulin in the filtrate was quantified using the Agilent 1100 series HPLC system (Agilent, Santa Clara, California, USA) composed of a quaternary pump, an autosampler, a column heater, and a DAD detector. Insulin was analyzed by C18 column (Zorbax, 3.5 μm, 4.6 mm × 150 mm, Agilent, USA) and detected at 214 nm. The mobile phase was acetonitrile and water with 0.1% of TFA in a gradient ratio from 10/90 to 100/0 for a 10 min run. The mobile phase was pumped at a flow rate of 1.0 ml/min. The column temperature was set to 20 °C. EE and LC in percentages were calculated using Eq. (1) and Eq. (2).

1 $$Entrapment\,efficiency\, (\%)=\left({1-Unencapsulated\, insulin}/{Total\, insulin}\right)\times 100\%$$

2 $$Loading\, content \left(\%\right)=\left(Weight \,of\, loaded\, insulin/Weight\, of\, NPs \right)\times 100\%$$

### Optimization of CS/insulin mass ratio and pH

Various ratios of CS/insulin ranging from 2.0 to 4.0 were tested to optimize the insulin NPs. Different amounts of CS solution were added during the preparation, whereas the insulin/TPP mixture was held constant. Insulin NPs were prepared at pH values ranging from 4.0 to 6.5 by carefully controlling the mixture's pH after all solutions were added (insulin, TPP and CS). The EE and particle sizes of the insulin nanoparticles at different pH values and CS/insulin mass ratios were evaluated to optimize the insulin NPs formation.

### Freeze drying of insulin NPs

The optimized insulin NPs were placed on an aluminum container and covered with a tissue tightened with some tapes. Subsequently, the tightened containers were put into a Labconco FreeZone freeze dryer equipped with tray dryers (Labconco, Kansas city, Missouri, USA). Temperature and vacuum pressure were set at −10 °C and 0.350 Torr for the first 2 h and 0 °C and 0.120 Torr for the remaining 22 h out of 24 h to obtain the dry insulin NPs.

### Spray drying of insulin NPs

A Buchi mini spray dryer B-290 (BÜCHI, Flawil, Switzerland) was used to generate the encapsulated insulin. The drying parameters selected were: temperature of 100 °C, feeding flow of 3 L/min, and airflow of 4 L/min.

### Fourier transform infrared spectroscopy

The insulin NPs before and after dehydration were characterized using FTIR-ATR spectroscopy. Dehydrated nanoparticles together with free insulin and chitosan were analyzed using Spectrum 100 FTIR spectrophotometer (PerkinElmer, Waltham, Massachusetts, USA) equipped with universal ATR sampling accessories (PerkinElmer, Waltham, Massachusetts, USA). Signal averages were obtained from 16 scans in the frequency range of 4000–600 cm^2^ at a resolution of 4 cm^2^.

### Scanning electron microscope (SEM)

The dry insulin NPs' morphology was evaluated by the SEM images of both freeze-dried and spray-dried insulin NPs taken by a Helios NanoLab 650 Focused Ion Beam—Scanning Electron Microscope (FIB-SEM) (FEI, Hillsboro, Oregon, USA). The main parameters used were a voltage 5 keV and a current of 30 mA.

### Reconstitution test

All dehydrated insulin NPs were redissolved in dd water. The particle sizes, PDI, EE, and LC were tested again using the same methods mentioned before to evaluate their quality after dehydration. The stability of the dehydrated insulin NPs was also measured by testing the properties of the NPs after a long time of storage. All NPs after dehydration were stored in the fridge for three months of this study. After three months storage the NPs were tested for their morphology particle sizes, PDI, EE and LC.

### Insulin protection efficacy

Five mL of redissolved NPs was diluted in 45 mL of digestive media containing simulated gastric fluid (pH 1.2, containing 1% of pepsin), intestinal fluid (pH 6.8, containing 1% of trypsin), or chymotrypsin solution (100 g/mL, in phosphate buffer, pH 7.8) to evaluate the insulin protects efficacy of the NPs after dehydration. They were incubated at a temperature of 37 °C with 100 rpm of stirring. 500 μL of the solution was collected in different time points, and the insulin concentration was determined using the HPLC.

### In vitro biological validation of insulin NPs

#### In vitro release profile of insulin-loaded NPs

The in vitro release behaviors of the fresh prepared and dehydrated insulin NPs were tested by dialysis sack method (cutoff molecular weight 100 kDa, Spectra Por Inc.). Fresh prepared and redissolved dry NPs were dialyzed in fluid with pH 2.5, pH 6.6, and pH 7.0 (0.1 M phosphate buffered saline, PBS), simulating the pH environments in the stomach, duodenum, and upper small intestine, respectively^28^. All samples were incubated at 37 °C with continuous shaking at 200 rpm. Five mL of the fluid outside the dialysis sack were withdrawn at the following times: 0.5, 1, 2, 3, 4 and 6 h, and the volume was immediately replenished with fresh dialysis fluid. The fluid was analyzed for insulin contamination by HPLC, and the release rate of insulin from nanoparticles was calculated from the ratio of released as free insulin to the total insulin encapsulated in nanoparticles (Eq. 3).

3 $$Insulin\, release\, rate \left(\%\right)=\left(Weight\, of \,released\, insulin/Weight\, of\, loaded \,insulin \right)\times 100\%$$

#### HepG2 cell culture

HepG2 cells^29^, human hepatocellular carcinoma cell line, were cultivated in petri dishes with 60 mm diameter using Dulbecco's Modified Eagle Medium (DMEM) containing 10% of fetal calf serum, 100 IU/mL of penicillin and 100 μg/mL of streptomycin. The culture was kept at the environment of 37 °C, 95% relative humidity with 5% CO_2_. For uptake assays, the HepG2 cells were seeded onto 8-well Nunc Lab-Tek chamber slide system (Thermo Fisher, NY, USA) at 1 × 10^5^ cells/ml. For cytotoxicity assays, they were seeded onto 96-well plates (Corning, NY, USA) with a density of 5 × 10^4^ cells/ml.

#### Cytotoxicity assay

The MTT test was used to assess the cytotoxicity of fresh prepared and dehydrated insulin NPs^30^. The HepG2 cells were seeded at a density of 5 × 10^4^ cells/mL in 96 well plates and cultivated for 7 days before the test. Insulin NPs were diluted to various concentration (50 to 500 μg/mL) in culture medium and then were given to the cells. After 24 h incubation, the cells were washed with PBS for three times and refreshed with medium containing 0.5 mg/ml of MTT for another 4 h incubation. The cytotoxicity was evaluated by measuring the enzymatic reduction of yellow tetrazolium MTT to purple formazan at 570 nm using Tecan infinite M200 pro spectrophotometer plate reader (Tecan, Männedorf, Switzerland).

#### NPs cellular uptake efficacy

The NPs cellar uptake efficacy was tested by confocal laser scanning microscope and flow cytometry analysis. Each well of Nunc Lab-Tek chamber slide system was treated with free FITC-insulin, FITC-insulin loaded NPs, and redissolved dehydrated FITC-insulin NPs at the same concentration of 25 μg/mL and incubated for 4 h. Cells were fixed with 4% paraformaldehyde after washing three times with PBS. The nuclei of the cells were stained with 4′,6-diamidino-2-phenylindole (DAPI). The localization of insulin was observed using a n Olympus FV1000 laser scanning/two-photon Confocal Microscope (Olympus, Shinjuku City, Tokyo, Japan). For flow cytometry analysis, free FITC-insulin, FITC-insulin loaded NPs, and redissolved dehydrated FITC-insulin NPs at the same concentration of 10 μg/mL were added into the 96-well plate seeded with HepG2 cells and incubated for 4 h. After 4 h incubation, cells were lifted and washed three times with FBS. 5 × 10^4^ cells per sample were analyzed by BD LSR II flow cytometer (BD, Franklin Lakes, New Jersey, United States).

### Statistical analysis

All values are expressed as Mean ± SD. Comparisons among all groups were evaluated using One-way ANOVA or t-test by IBM SPSS Statistics 26 for Mac (IBM, Endicott, New York, USA), and *p* < 0.05 was considered to be statistically significant.

## Conclusions

This study demonstrates the flexibility and capability of spray drying to dehydrate crosslinked chitosan/TPP/insulin NPs, which showed superior reconstitution ability and higher loading content than the standard freeze-drying method with bulking agent or cryoprotectant. The optimized insulin NPs resulted in 318 nm of mean particle size and 99.4% encapsulation efficiency. After dehydration, the SEM and FTIR results suggested that the spherical structure was maintained only for the NPs spray-dried with and without mannitol and freeze-dried with mannitol, but the freeze-dried NPs without mannitol broke down during the dehydration process. In the reconstitution ability test, insulin NPs spray dried without mannitol showed the smallest mean particle sizes and highest loading content when redissolved. The release behaviors of all these dehydrated NPs showed that they could all fast release in solution with pH = 2.5 and pH = 7, while they were very stable in the solution with a pH of 6.5. The NPs spray dried without mannitol showed the fastest release compared with other redissolved dehydrated NPs. This result was consistent with the results observed in the cellular uptake test as the NPs spray dried without mannitol almost fully maintained cellular uptake efficiency of the fresh prepared NPs. These results suggested that the dry insulin NPs produced by spray drying with no mannitol were most suitable for further processing into other anhydrous dosage forms such as oral tablets or bioadhesive films.

## Supplementary Information

Supplementary Information.

## Data Availability

The datasets generated and/or analysed during the current study are not publicly available due Intellectual Property issues, but are available from the corresponding author on reasonable request.
