# Supplementary material for: Production of high loading insulin nanoparticles suitable for oral delivery by spray drying and freeze drying techniques
Source: Sci Rep. 2022 Jun 15;12:9949. doi: 10.1038/s41598-022-13092-6 (PMC9200718; doi:10.1038/s41598-022-13092-6)
Supplement: Supplementary file 1 — Supplementary Information. [file 41598_2022_13092_MOESM1_ESM.docx]

**Production of high loading insulin nanoparticles suitable for powder delivery by spray drying technique**

Yigong Guo ^1^, Alberto Baldelli ^1^ , Farahnaz Fathordoobady^1^_,_ Anika Singh^2^, David Kitts^1^ and Anubhav Pratap-Singh^1^

^1^Faculty of Land and Food Systems (LFS), University of British Columbia, Vancouver Campus 213-2205 East Mall, Vancouver, BC Canada V6T 1Z4

^2^ Natural Health and Food Products Research Group, Centre for Applied Research & Innovation (CARI), British Columbia Institute of Technology, 4355 Mathissi Pl, Burnaby, BC, Canada V5G 4S8

**
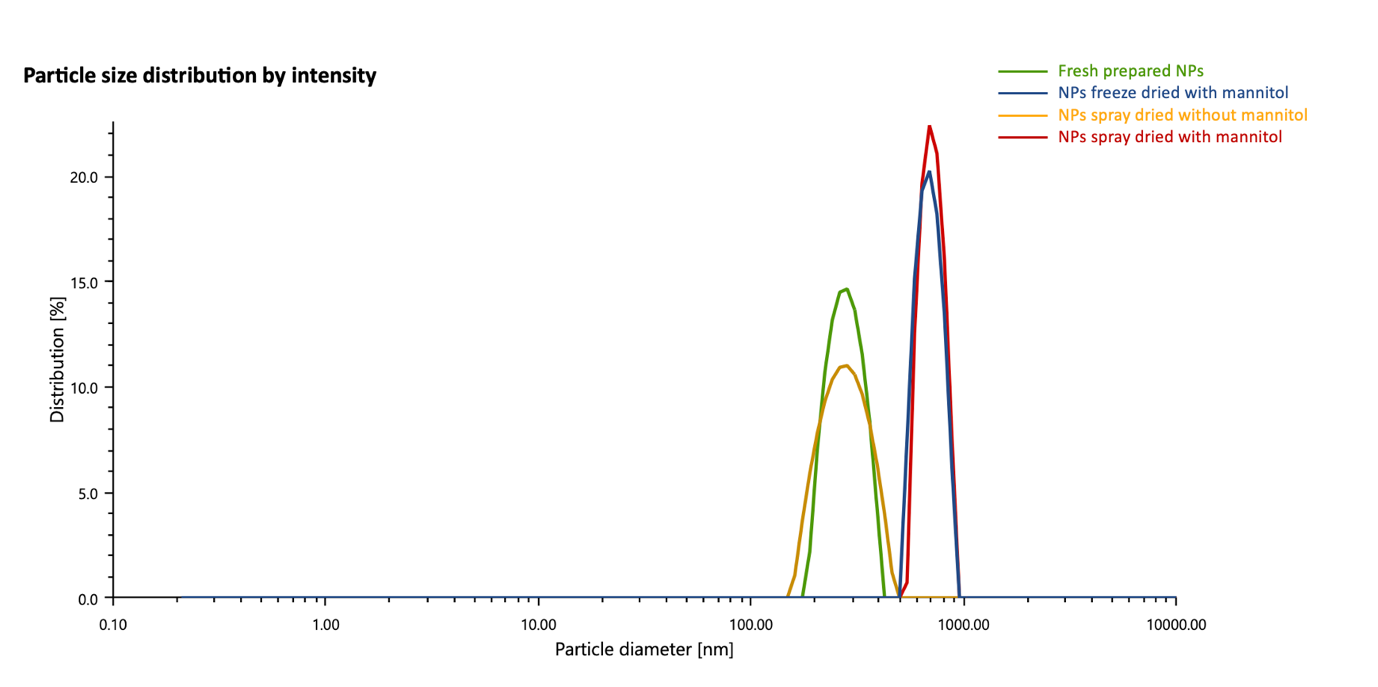
**

**Supplementary Figure S1.** Particle size distribution of reconstituted insulin NPs compared with fresh prepared insulin NPs

**
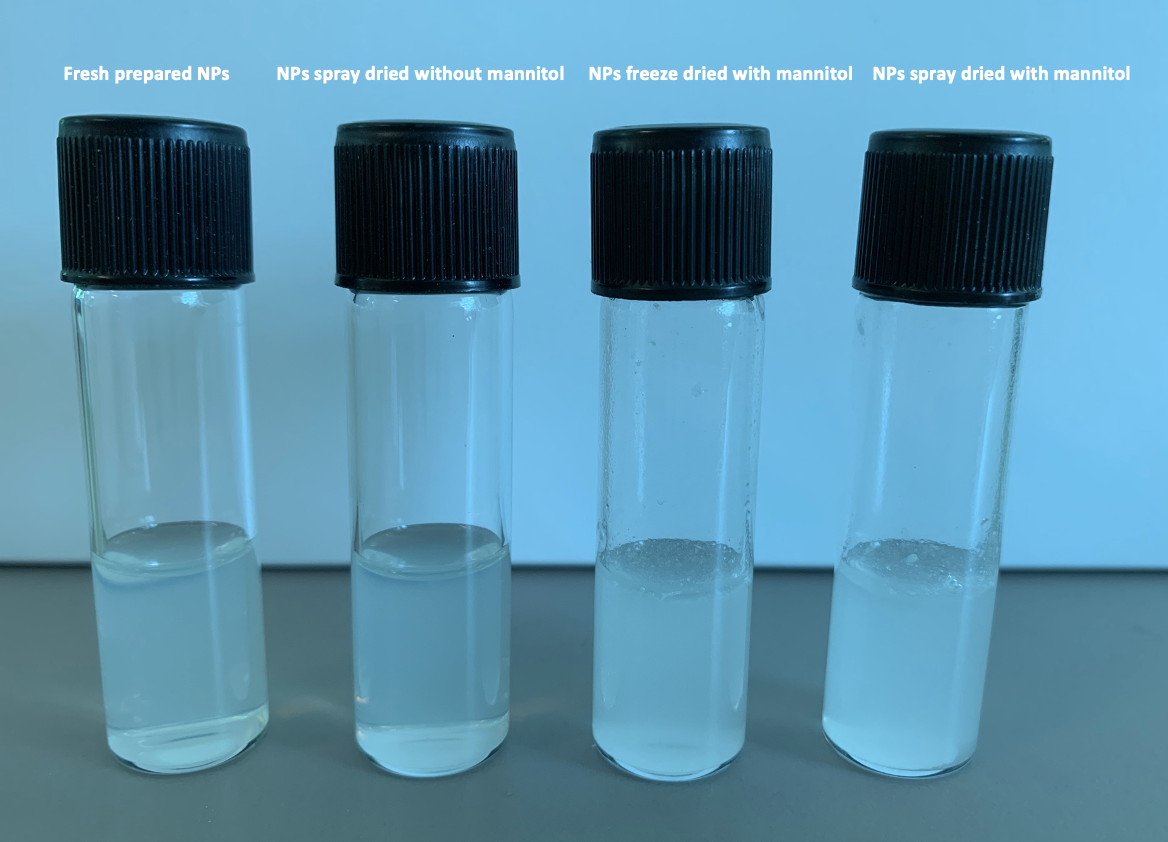
**

**Supplementary Figure S2.**  Image of reconstituted insulin NPs after three months


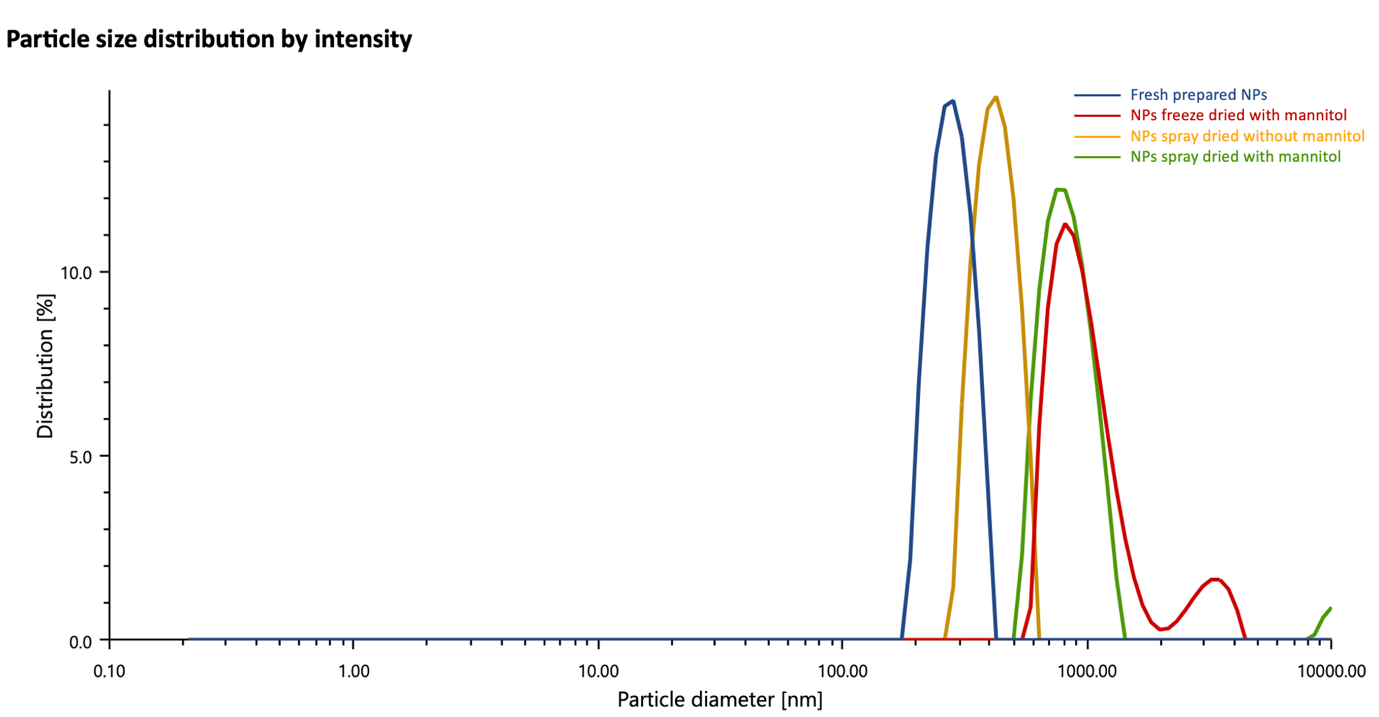


**Supplementary Figure S3.** Particle size distribution of reconstituted insulin NPs after three-month storage compared with fresh prepared insulin NPs


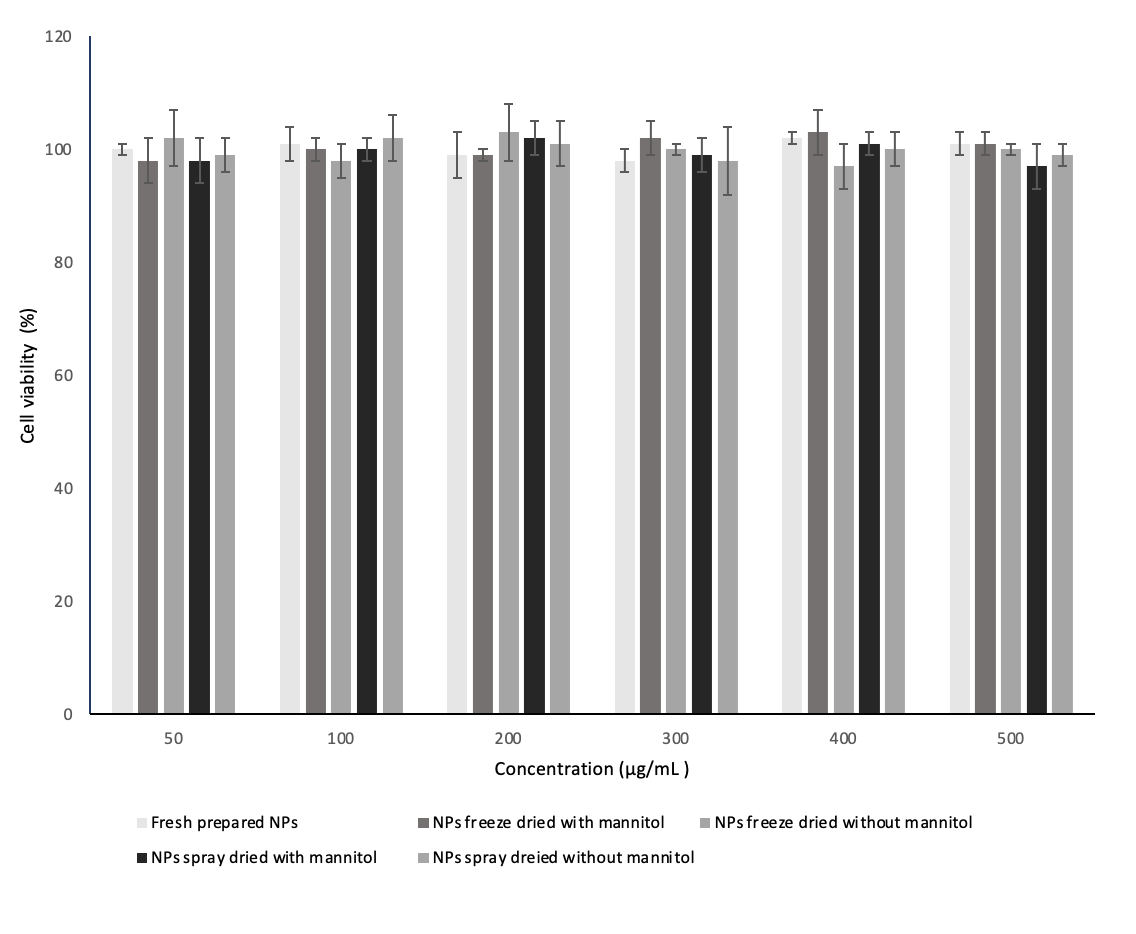


**Supplementary Figure S4.** Cytotoxicity of different insulin NPs
